# RWP-RK Domain 3 (OsRKD3) induces somatic embryogenesis in black rice

**DOI:** 10.1186/s12870-023-04220-z

**Published:** 2023-04-19

**Authors:** Yekti Asih Purwestri, Yang-Seok Lee, Cathal Meehan, Windi Mose, Febri Adi Susanto, Putri Wijayanti, Anisa Nazera Fauzia, Tri Rini Nuringtyas, Nosheen Hussain, Hadi Lanang Putra, Jose Gutierrez-Marcos

**Affiliations:** 1 Research Center for Biotechnology, Universitas Gadjah Mada Jl. Teknika Utara, Depok, Sleman, Yogyakarta, Indonesia 55281; 2 Department of Tropical Biology, Faculty of Biology, Universitas Gadjah Mada Jl. Teknika Selatan, Sekip Utara, Yogyakarta, Indonesia 55281; 3 School of Life Sciences, University of Warwick, Coventry, CV4 7AL UK

**Keywords:** Cell reprogramming, Tissue regeneration, Somatic embryogenesis, Transformation, Black rice

## Abstract

**Background:**

Plants have the unique capability to form embryos from both gametes and somatic cells, with the latter process known as somatic embryogenesis. Somatic embryogenesis (SE) can be induced by exposing plant tissues to exogenous growth regulators or by the ectopic activation of embryogenic transcription factors. Recent studies have revealed that a discrete group of *RWP-RK* DOMAIN-CONTAINING PROTEIN (*RKD*) transcription factors act as key regulators of germ cell differentiation and embryo development in land plants. The ectopic overexpression of reproductive *RKDs* is associated with increased cellular proliferation and the formation of somatic embryo-like structures that bypass the need for exogenous growth regulators. However, the precise molecular mechanisms implicated in the induction of somatic embryogenesis by RKD transcription factors remains unknown.

**Results:**

In silico analyses have identified a rice RWP-RK transcription factor, named Oryza sativa RKD3 (OsRKD3), which is closely related to Arabidopsis thaliana RKD4 (AtRKD4) and Marchantia polymorpha RKD (MpRKD) proteins. Our study demonstrates that the ectopic overexpression of OsRKD3, which is expressed preferentially in reproductive tissues, can trigger the formation of somatic embryos in an Indonesian black rice landrace (Cempo Ireng) that is normally resistant to somatic embryogenesis. By analyzing the transcriptome of induced tissue, we identified 5,991 genes that exhibit differential expression in response to OsRKD3 induction. Among these genes, 50% were up-regulated while the other half were down-regulated. Notably, approximately 37.5% of the up-regulated genes contained a sequence motif in their promoter region, which was also observed in RKD targets from Arabidopsis. Furthermore, OsRKD3 was shown to mediate the transcriptional activation of a discrete gene network, which includes several transcription factors such as APETALA 2-like (AP2-like)/ETHYLENE RESPONSE FACTOR (ERF), MYB and CONSTANS-like (COL), and chromatin remodeling factors associated with hormone signal transduction, stress responses and post-embryonic pathways.

**Conclusions:**

Our data show that *OsRKD3* modulates an extensive gene network and its activation is associated with the initiation of a somatic embryonic program that facilitates genetic transformation in black rice. These findings hold substantial promise for improving crop productivity and advancing agricultural practices in black rice.

**Supplementary Information:**

The online version contains supplementary material available at 10.1186/s12870-023-04220-z.

## Background

The germline in animals is established from primordial germ cells during early embryogenesis, however, plants germ cells are produced later in development, representing the reduced haploid phase, through the reprogramming of diploid somatic cells. While both plants and animals can form embryos from zygotes (zygotic embryogenesis; ZE) upon gamete fusion, plants alone, have the additional capability of forming embryos from differentiated somatic cells (somatic embryogenesis; SE). SE represents a classic model of totipotency where somatic cells of the plant retain sufficient developmental plasticity to give rise to all the cell types that are required to form an entire new organism.

SE in plants can be stimulated by exogenous plant growth regulators (PGRs) – such as phytohormones (auxins and cytokinins) and this response can be enhanced by stress (temperature, heavy metals or osmotic shock) [1, 2], a property that has been exploited for clonal propagation [3–5] and genetic manipulation [6–11]. However, not all plants are susceptible to SE via these methods, representing a significant caveat for the propagation of agronomically important elite lines, and so other approaches must be sought to overcome these current limitations. Studies in Arabidopsis have revealed a few transcription factors (TFs) that are necessary for ZE that when ectopically activated in somatic tissues can also induce the formation of embryonic structures [6, 12–15]. Several embryogenesis factors are currently utilized to improve the efficiency of genetic modification – overexpression of BABY BOOM (BBM) and WUSCHEL 2 (WUS2) have been shown to increase the transformation efficiency of several grass species [16, 17]. Furthermore, the overexpression of fusion proteins consisting of GROWTH REGULATING FACTOR 4-GROWTH REGULATING FACTOR INTERACTING FACTOR 1 (GRF4-GIF1) has been demonstrated to enhance the efficiency of both genetic transformation and genome editing in wheat [18]. The *RWP-RK* DOMAIN-CONTAINING PROTEIN (*RKD*) gene family is of particular interest because its member genes are widely conserved in a range of plant species [12, 19–23]. The *RKD* gene family is divided into two major groups – one comprising genes primarily expressed in reproductive organs during germline development and early embryogenesis [11, 12, 24], and the other comprising genes that are expressed during symbiosis and/or in response to nitrogen deficiency [19, 25]. Notably, reproductive RKD transcription factors are evolutionarily conserved and play a critical role in the regulation of germ cell differentiation in land plants [21, 26]. The ectopic expression of *Arabidopsis* RKD4 (*AtRKD4*) results in increased cellular proliferation and the formation of somatic embryo-like structures [11]. Because these transcription factors are evolutionarily conserved in plants, we sought to investigate their molecular function and assess their potential use as molecular tools to engineer SE in species recalcitrant to in vitro propagation technology. The black rice landrace *Oryza sativa* L. cv. Cempo Ireng, which is cultivated by small-hold farmers in Indonesia, has potent nutraceutical properties due to the accumulation of secondary metabolites in its seeds [27–29]. As with many other rice landraces, Cempo Ireng is recalcitrant to somatic embryo induction using exogenous plant growth regulators, thus the efficiency of genetic transformation using conventional tissue culture is low (Susanto et al., 2020). As proof of concept, we identified reproductive RKD in rice (OsRKD3) and found the ectopic expression of this gene under the control of a chemically inducible system led to the formation of somatic embryos and an increase in transformation in black rice. We also found that OsRKD3 targets a discrete transcriptional network associated with the activation of an embryonic program and the repression of vegetative development. Notably, some components of this gene network are also activated by RKD4 in *Arabidopsis*. Collectively, our work has uncovered a mechanism(s) by which RKD transcription factors induce the formation of somatic embryos in plants, thereby facilitating the genetic manipulation and clonal propagation of neglected yet valuable crop species.

## Results

### Induction of somatic embryogenesis by *OsRKD3* in black rice

**Fig. 1:**
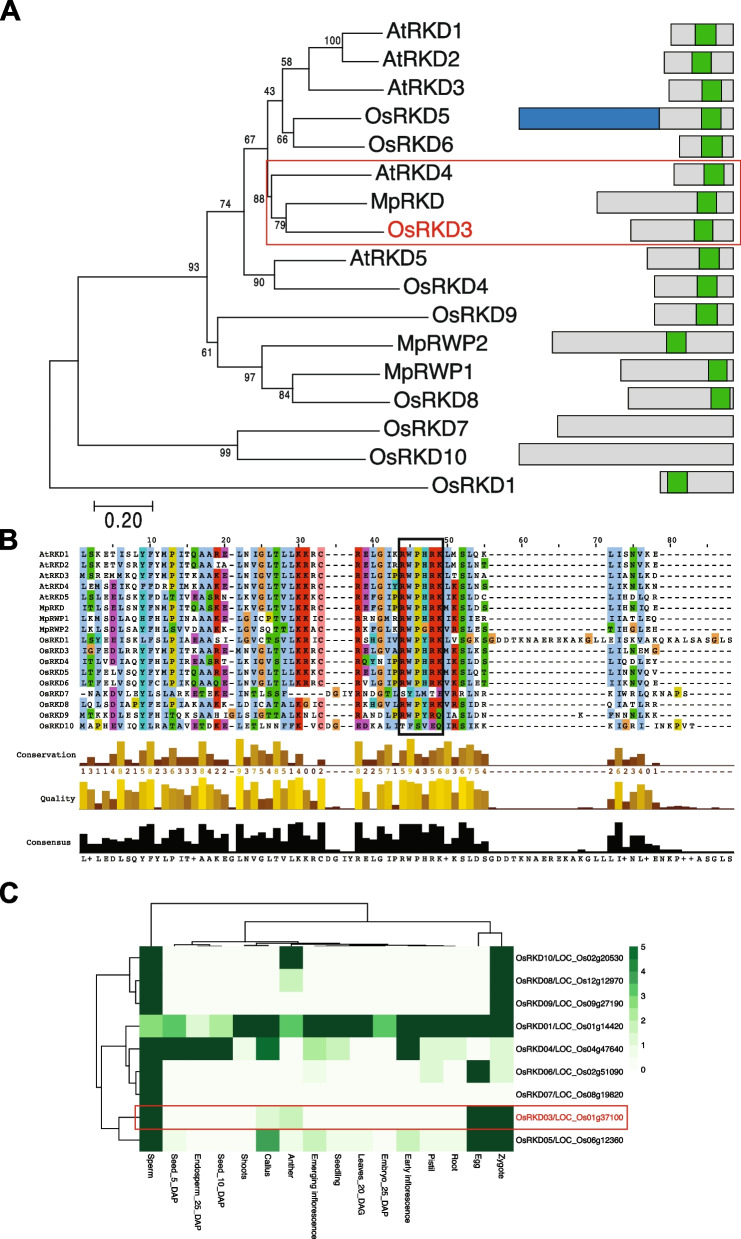
OsRKD3 is closely related to embryonic RKDs. **A** Phylogenetic tree of RWP-RK proteins. Left panel; Phylogenetic tree computed with MEGA7, representing the evolutionary history, was inferred using the minimum evolution method. An optimal tree with the sum of branch length = 8.65634175 is shown. The ME tree was generated using the Close-Neighbour-Interchange (CNI) algorithm at a search level of 1. The percentage of replicate trees in which the associated taxa clustered together in the bootstrap test (500 replicates) are shown next to the branches. The analysis involved 17 amino acid sequences. All positions containing gaps and missing data were eliminated. Right panel: Schematic diagrams for structures of RWP-RK proteins. Green boxes represented RWP-RK domain. Blue box on OsRKD5 represented tetratricopeptide repeats (TPR) and pentatricopeptide repeats (PPR). Red box highlights AtRKD4, MpRKD and OsRKD3, which group together in the phylogenetic tree. Os, *Oryza sativa*; At, *Arabidopsis thaliana*; Mp, *Marchantia polymorpha*. **B** Protein alignment of RWP-RK domains. The alignment computed by MAFFT using Jalview. A black coloured box represented the RWP-RK conserved motif. **C** Expression profiling, as normalised FPKM RNA-seq reads, of *RKD* genes in various rice organs. Red box indicates the expression profile for *OsRKD3*. **D** Expression profile of OsRKD3 in different reproductive tissues. RNA-seq data was obtained from the rice genome annotation project (http://rice.uga.edu/expression.shtml) and the rice RNA-seq database (http://ipf.sustech.edu.cn/pub/ricerna/)

To identify *RKD* genes with the potential to induce the formation of somatic embryos in black rice, we performed protein alignments with RKD proteins from three evolutionarily distant plant species (*Marchantia polymorpha, Arabidopsis thaliana* and *Oryza sativa*) and conducted a phylogenetic analysis. We found that with the exception of OsRKD7 (*LOC_Os08g19820)* and OsRKD10 (*LOC_Os02g20530)*, rice RKD proteins have a single RWP-RK domain. Of these, OsRKD3 (*LOC_Os01g37100)* was the most closely related to AtRKD4 and MpRKD proteins (Fig. 1A-B), which have been shown to be necessary for embryo and germline development [11, 21, 30]. Transcriptome analysis revealed that *OsRKD3* was expressed primarily in gametes and zygotes, while other RKD encoding genes were expressed constitutively in vegetative tissues (*OsRKD1* (*LOC_Os01g14420)*, *OsRKD5* (*LOC_Os06g12360)* and *OsRKD6* (*LOC_Os02g51090))* or in developing seeds (*OsRKD4* (*LOC_Os04g47640), OsRKD8* (*LOC_Os12g12970)* and *OsRKD10* (*LOC_Os02g20530)*) (Fig. 1C).

**Fig. 2:**
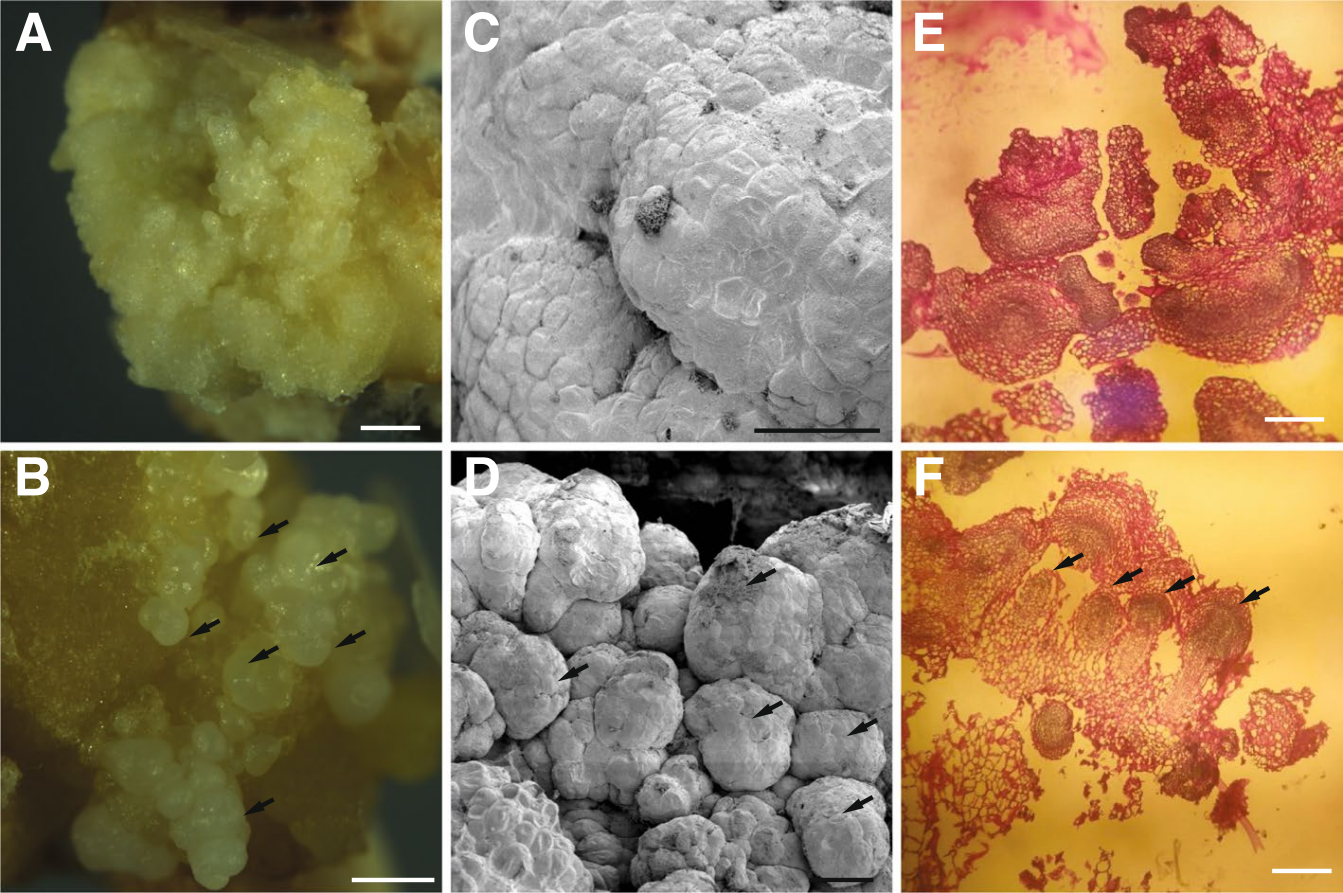
Induction of somatic embryos in black rice upon ectopic expression of OsRKD3. Morphology of calli transformed with *Agrobacterium tumefaciens* carrying the indOsRKD3 construct, grown in N6 media containing mock or DEX (5 days) and transferred to chemical free N6 media (5 days) for analysis. Representative images of mock-treated (**A**) and DEX-treated calli (**B**). Scanning electron microscopy (SEM) images of mock-treated (**C**) and DEX-treated (**D**) calli. Histological analysis of mock-treated (**E**) and DEX-treated (**F**) calli stained with PAS-Haematoxylin. Black arrows indicate meristematic-like structures. Scale bars, 100 μm

**Fig. 3:**
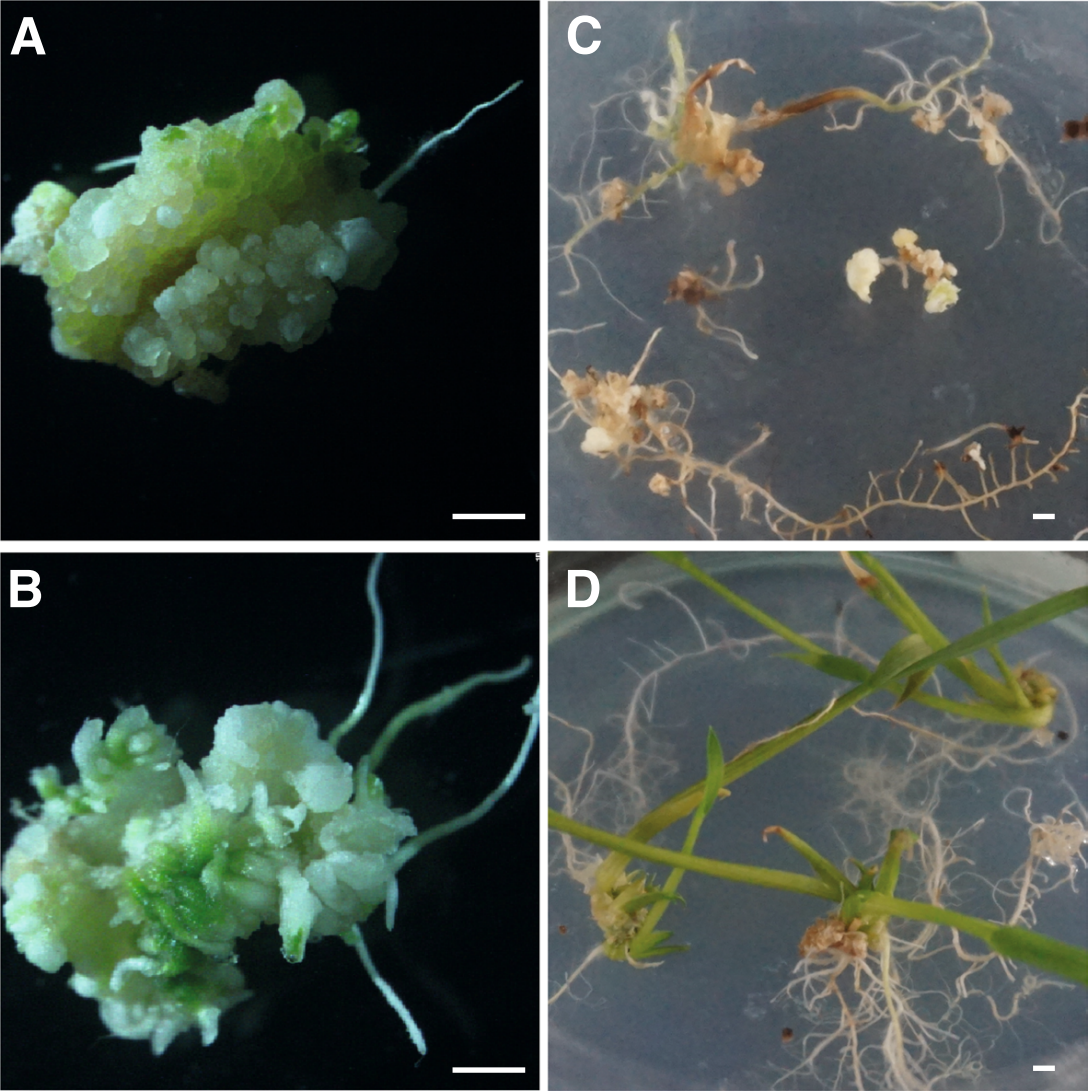
OsRKD3 induces embryogenesis in the absence of exogenous phytohormones. Representative images of indOsRKD3 transformed immature embryos tissue grown in phytohormone-free media. **A** Calli from mock-treated sample. **B** Shoot arising from calli of DEX-treated sample. **C** Lack of shoot development in mock treated calli. **D** Abundant shoots developing in DEX-treated samples. Scale bars, 100 μm

**Fig. 4:**
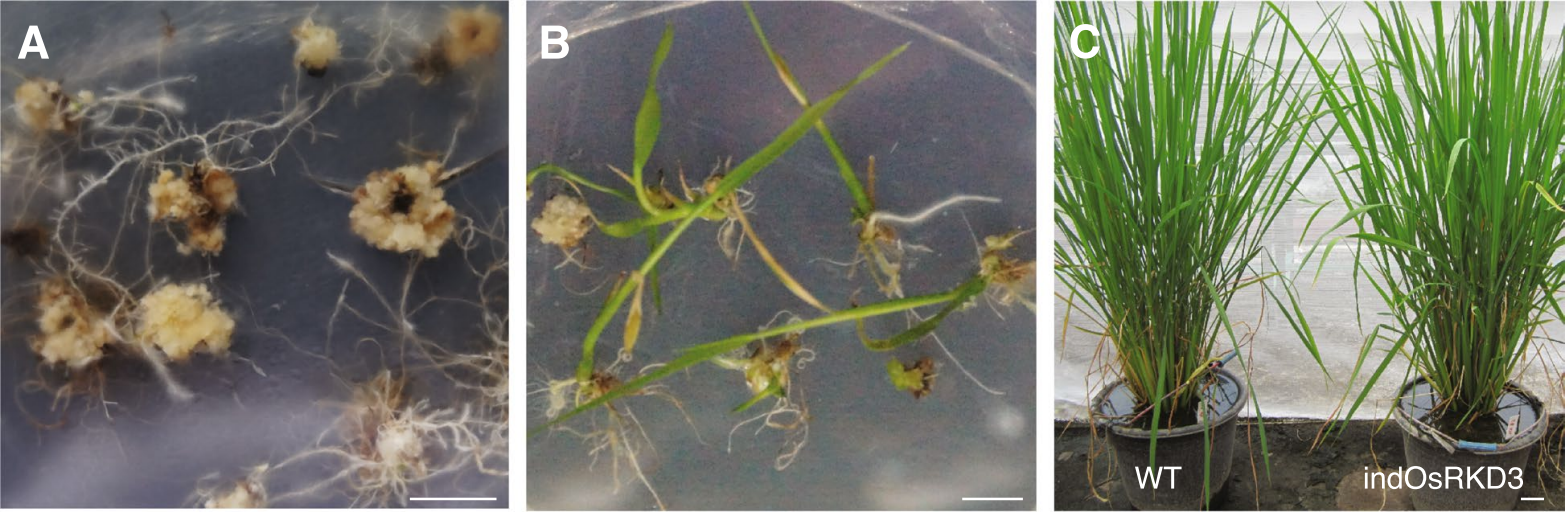
High frequency regeneration of black rice plants using indOsRKD3. Representative images of indOsRKD3-transformed immature embryos incubated with a mock solution (1% shoots; n > 200 calli) (**A**) or incubated for five days with a 20 μM DEX solution (98%; n > 200 calli) (**B**). Similarities in growth between wild-type (WT) and transgenic indOsRKD3 plants grown in soil under glasshouse conditions (**C**). Scale bars, 1 cm, 1 cm and 5 cm, respectively

To define the molecular function of *OsRKD3* and its capacity for inducing somatic embryogenesis, we synthesised a *OsRKD3* (*synOsRKD3*) gene that was subcloned into a chemically-inducible binary vector [31], named indOsRKD3, which enables the nuclear translocation of *synOsRKD3* upon exposure to dexamethasone (DEX). We infected calli from black rice immature embryos with *Agrobacterium* carrying indOsRKD3 and transferred them to media containing hygromycin. Hygromycin-selected calli were then transferred to DEX or mock containing medium and after eight days transferred to a medium free of chemical inducer or exogenous phytohormones. We found that none of the mock-treated calli (*n* = 50) was able to develop somatic embryo structures (Fig. 2A), whereas all DEX-treated indOsRKD3 calli (*n* = 50) formed somatic embryo structures (Fig. 2B). To assess the morphology of these somatic embryo structures in more detail, we used Scanning Electron Microscopy (SEM), which revealed that cells of untreated calli were uniformly distributed on the surface (Fig. 2C), while treated calli formed dense cellular aggregates resembling somatic embryo structures (Fig. 2D). Histological analysis showed that only DEX-treated indOsRKD3 calli displayed abundant meristematic-like structures typically found in somatic embryos (Fig. 2E-F). We treated calli with Sudan Red 7B – a dye that stains triacylglycerols [32] and that accumulates in embryos and somatic embryo structures in plants [33]. We found that compared to untransformed calli, mock-treated indOsRKD3 calli had weak Sudan Red 7B staining but when treated with DEX indOsRKD3 calli showed a strong increase in staining (21.7-fold) (Fig. S1). To determine the capacity of these somatic embryos to differentiate into new organs, we transferred these calli to culture media lacking phytohormones. We found that mock-treated indOsRKD3 calli formed poorly developed roots and shoots (Fig. 3 A, C), while DEX-treated indOsRKD3 calli made abundant roots and shoots (Fig. 3 B, D). To determine if the formation of roots and shoots in these samples was a direct consequence of the ectopic induction of *OsRKD3,* we infected immature embryos with *Agrobacterium* carrying the indOsRKD3 construct in phytohormone-free media with or without DEX. We found that ind*OsRKD3* treated calli grown in media lacking DEX did not produce visible shoots (Fig. 4A), but those grown in media with DEX produced abundant shoots (Fig. 4B-C) that when transferred to soil grew to maturity and produced seeds. To test if these plants had originated from cells that were stably transformed with the indOsRKD3 construct, we used mature leaves from selected lines for molecular genotyping and confirmed that all samples contained the stable integration of the indOsRKD3 transgene (Fig. S2A). The transformation frequency for Agrobacterium-mediated transformation was estimated with either indOSRKD3 or an empty vector. The results indicated that indOSRKD3 significantly increased the frequency of transformation, with an increase ranging from 2.9-fold in mock-treated samples to 23.5-fold in dexamethasone-treated samples (Table S1). Collectively, these results suggest that ectopic expression of *OsRKD3* induces the formation of somatic embryos and increases the efficiency of genetic transformation in black rice.

### *OsRKD3* activates a transcriptional cascade that is associated with the formation of SE

**Fig. 5:**
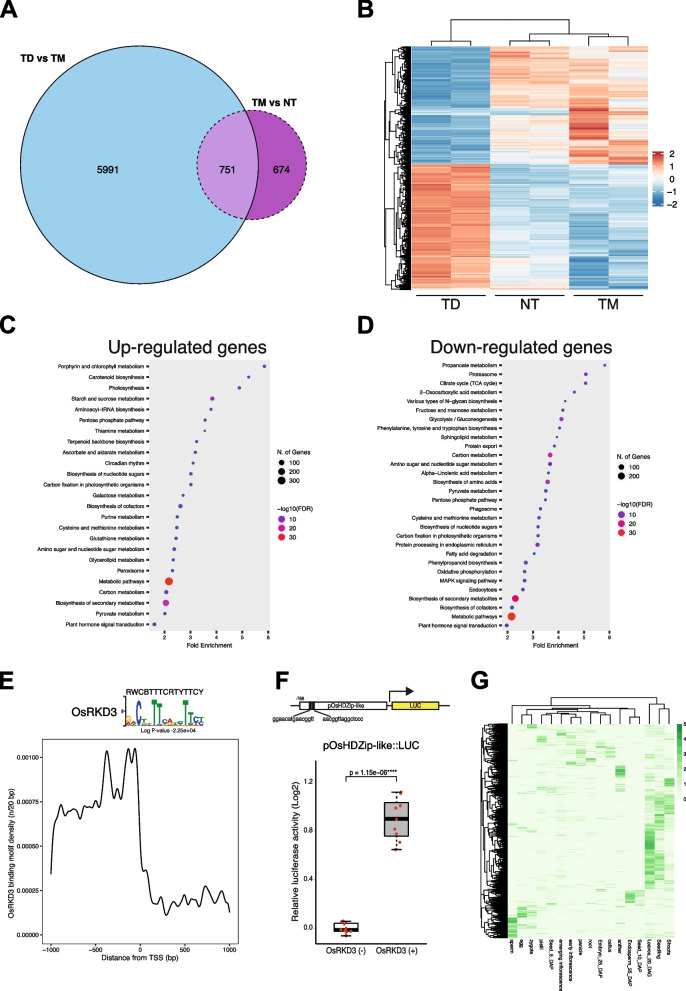
Transcriptional changes directed by OsRKD3 induction. **A** Venn diagram showing the overlap between differentially expressed genes (DEGs) in OsRKD3 induced and OsRKD3 leaked samples. Blue coloured circle represents DEGs between indOsRKD3 DEX (TD) and indOsRKD3 mock (TM) treatments; purple coloured circle indicates DEGs between indOsRKD3 mock (TM) and non-transgenic (NT) treatments. **B** Heatmap showing the transcriptional changes of differentially expressed genes found in indOsRKD3 transgenic and non-transgenic plants (two biological replicates per sample). **C, D** Gene Ontology (GO) analysis showing gene sets enriched in the differentially upregulated (**C**) and downregulated (**D**) categories. (**E**) Distribution of RKD-binding motifs in the upstream regions of OsRKD3 upregulated DEGs. Regulatory regions were defined as 1000 bp upstream of the transcription start site (TSS) and 1000 bp downstream of TSS. RKD motif enrichment and statistical significance was determined using HOMER v4.11. RKD motif density was plotted using a bin size of 20 bp. **F** Functional analysis of cis-regulatory motifs identified in OsRKD3 targets. The panel represents the promoter activity of HDZip-like/OsHox2 in *Arabidopsis* protoplasts upon OsRKD3 induction. Top panel: a schematic diagram showing the construct designed to determine promoter activity using a luciferase reporter. Black boxes, RKD-binding motifs. Bottom panel: quantitative measurements of luciferase activity of the promoter. A t-test was performed; ****p < 0.0001. **G** Heatmap showing the expression, as nomalised FPKM reads, in different rice tissues of OsRKD3 upregulated DEGs that contain RKD sequence motifs

To ascertain the molecular changes associated with *OsRKD3-*induced formation of SEs in black rice, we performed a genome-wide transcriptome analysis by RNA-seq*.* For this analysis, we used calli from mature embryos isolated from indOsRKD3 and wild-type siblings plants. We could not detect *synOsRKD3* in non-transgenic mock (NT). By contrast, *synOsRKD3* was weakly activated in transgenic mock (TM), and strongly activated (> eightfold) in DEX treated transgenic samples (TD) (Fig. S2B). We conducted pairwise comparisons between the transcriptomes of transgenic DEX-treated (TD) and transgenic mock-treated (TM) and found 6,742 differentially expressed genes (DEGs) (padj < 0.001, |log2FC|> 1; Supporting Table S2). To discount the possible effects of the basal expression of *synOSRKD3*, we compared the transcriptomes of transgenic mock-treated (TM) samples and non-transgenic mock-treated (NT) samples, which identified 1,425 DEGs (padj < 0.001, |log2FC|> 1; Supporting Table S3). When we overlapped these datasets, we found 751 genes that were misregulated in response to the mock treatment and thus were excluded from further analyses (Supporting Tables S4). We found that out of the 5,991 genes misregulated in response to *OsRKD3* induction, half (2,928 genes) were down-regulated while the other half (3,063 genes) were up-regulated (Fig. 5A-B). In wild type plants, the genes upregulated upon *OsRKD3* induction were preferentially expressed in early developing seeds, embryos, and inflorescences, while the downregulated DEGs were primarily expressed in vegetative tissues: shoots, leaves and seedlings (Figs. S3 and S4). Gene Ontology (GO) analysis revealed that upregulated DEGs were involved primarily in photosynthesis and circadian rhythm (Fig. 5C, D). By contrast, downregulated DEGs were predominantly associated with proteasome and phosphorylation pathways, but also with MAPK signaling pathway (Fig. 5C, D). We also found a large number of transcription factors (TFs) misregulated in response to *OsRKD3* induction, amongst which the AP2, MYB, and CONSTANS-like transcription factors were notably enriched and BABY BOOM 1-LIKE/AP2-EREBP (*LOC_Os02g51300*) was included in up-regulated TFs as well (Supporting Table S5-S6). In addition, we found that embryogenesis and chromatin remodelling-associated genes were also strongly misregulated upon *OsRKD3* induction (Supporting Table S5). Collectively, these data suggest that *OsRKD3* plays a dual role in the activation of the embryonic program and in the repression of the vegetative program in black rice.

### *OsRKD3* acts primarily as a positive regulator of transcription

**Fig. 6:**
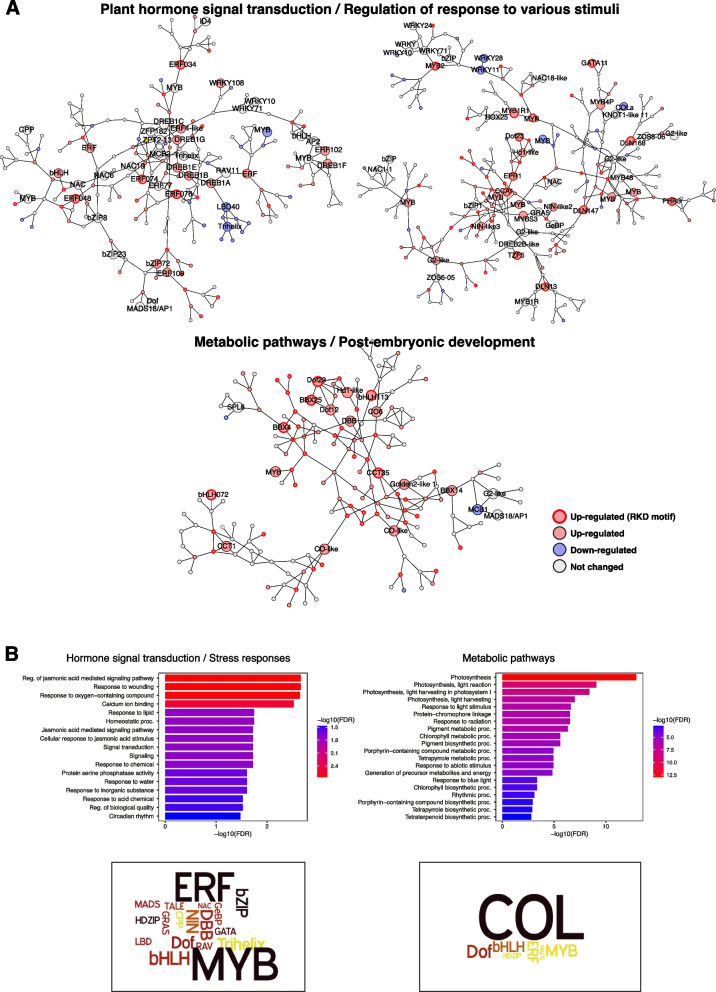
OsRKD3 controls a discrete transcriptional gene network. **A** Co-expression network of upregulated DEGs identified annotated by differential expression. Nodes (genes) and edges (co-expression relationships) are spatially arranged with a profuse force-directed layout, and clustered according to spatial separation. Red coloured circles represented up-regulated genes, blue-coloured circles represented down-regulated genes, and grey-coloured circles represented non-changed genes. Of red-coloured circles, red border circles represented up-regulated genes that have RKD motifs in their promoter. **B** GO enrichment (top panels) and TFs enrichment (bottom panels) of co-expression nodes for plant hormone signal transduction/response to stimulus (a left panels) and metabolic pathways/post-embryonic development (right panels). DEGs co-expression network are plotted with a -log10 transformation

To delineate the components of the gene network that may be directly regulated by *OsRKD3,* we searched for sequence binding motifs in *OsRKD3* upregulated DEGs by scanning these sequences for RKD motifs identified in Arabidopsis [34]. We found that 37.5% of the upregulated DEGs (1,150/3,063) contained a conserved RKD-like binding motif (RWCBTTTCRTYTTCY) within their 1 kb promoter region (Supporting Table S7; Fig. 5E). These putative binding motifs were found primarily in proximity to transcription start sites (TSS) of upregulated DEGs (Fig. 5E). To check whether the putative binding motif is recognised by OsRKD3, we fused the upstream promoter region of the transcription factor gene *Homeodomain leucine zipper*/*OsHox2* (*LOC_Os06g04870*), which contains two putative OsRKD3 binding motifs, to the firefly luciferase (LUC) reporter gene (*pOsHDZip-like::LUC*) (Fig. 5F). We found that co-transfection of Arabidopsis protoplasts with *pOsHDZip-like::LUC* and *pUBI::synOsRKD3* resulted in a significant increase in LUC reporter expression (Student t-test, *p* < 0.001). Using publicly available transcriptome datasets, we investigated the expression profile of genes carrying OsRKD3 binding motifs. We found that *OsRKD3* upregulated DEGs carrying an RKD binding motif were preferentially expressed in egg cells and zygotes (8.17%; 94/1,150), and sperm cells (9.83%; 113/1,150) (Supporting Tables S9; Fig. 5G). Notably, some of the genes containing a OsRKD3 binding motif and upregulated after OsRKD3 ectopic expression encoded transcription factors, such as *Oryza sativa basic helix-loop-helix 148* (*OsbHLH148, LOC_Os03g53020), OsMYB2 (LOC_Os03g20090), OsGATA11 (LOC_Os02g12790), OsGRAS8 (LOC_Os02g44370), PHOSPHATE STARVATION RESPONSE 3 (OsPHR3, LOC_Os02g04640), NAM, ATAF1-2, AND CUC2 (NAC) 14 (OsNAC14, LOC_Os01g48446), DNA-binding with one zinc finger 23 (OsDof23, LOC_Os07g48570), APETALA2/ethylene-responsive element binding factor 48 (OsERF048, LOC_Os08g31580), OsWRKY74 (LOC_Os09g16510), B-box protein 4 (OsBBX4, LOC_Os02g39360), BEL1-like homeodomain 1 (OsBLH1, LOC_Os12g06340), Homeodomain leucine zipper (OsHDZip)/OsHox2 (LOC_Os06g04870), OsHox16 (LOC_Os02g49700), Indeterminate domain 10 (OsIDD10, LOC_Os04g47860), OsMADS37 (LOC_Os08g41960), Zince-finger homeodomain protein 3 (OsZHD3, LOC_Os12g10630)*, and *chromatin-remodeling factors, Rice histone deacetylase 10 (OsHDAC10, LOC_Os12g08220*) and *Jumonji C domain-containing histone demethylase 702 (OsJMJ702, LOC_Os12g18150*). To dissect the transcriptional cascade regulated by *OsRKD3* we conducted a network analysis using upregulated TFs that contained a conserved RKD-like binding motif within their 1 kb promoter region. We identified three distinct gene networks, which have been implicated in hormone signal transduction, stress responses, and metabolic pathways (Fig. 6A; Supporting Table S8-9). These networks comprised 252 upregulated genes, 167 downregulated genes, and were associated with the expression of transcription factors belonging to the ten different families (Fig. 6A; Supporting Table S8-9). Further examination of these co-expression networks revealed that the hormone signalling and stress response network was enriched for AP2/ERF and MYB transcription factors, and associated with response to wounding and Jasmonic-acid mediated responses (Fig. 6B; Supporting Table S9). On the other hand, the gene network implicated in metabolic pathways was enriched for CONSTANS-like (COL) transcription factors.

Taken together our data suggest that *OsRKD3* activates a group of transcriptional and chromatin regulators associated with the activation of an embryonic program and the formation of embryo-like structures in somatic cells.

## Discussion

The regeneration of whole plants from differentiated somatic tissues has been of paramount importance for the advancement of plant biotechnology and crop improvement. The regeneration properties of tissue explants differ widely between species, while the molecular factors underpinning these differences remain elusive [35]. The application of stress, which results in the disruption of cell–cell communication, is thought to be an important trigger for the initiation of de novo organogenesis and the regeneration in plants [36]. Genetic analyses have revealed that the AP2/ERF-type transcriptional factors WOUND-INDUCED DEDIFFERENTIATION1-4 (*WIND1*-4) are induced upon mechanical damage and promote the formation of calli at wound sites [8, 37]. Wounding also induces the accumulation of endogenous phytohormones, primarily auxin and cytokinin, which are associated with changes in cell cycle progression and the differentiation state of cells in damaged tissues [38]. Our data support the view that Jasmonate-mediated signalling responses are implicated in plant regeneration [39]. Phytohormones also play a critical role in developmental patterning during tissue regeneration in culture, which is thought to result from the interaction between hormone signalling and cell-identity pathways [40, 41]. These initial triggers are believed to cause the dynamic epigenetic and transcriptional changes associated with the acquisition of competency that is necessary to convert the fate of differentiated tissues [42].

An alternative method to de novo organogenesis is the induction of somatic embryogenesis, which can be achieved using exogenous phytohormones or through the ectopic expression of some embryogenic transcription factors [6, 12–14, 43]. Among these, the RWP-RK motif-containing transcription factors (RKD) are of particular interest because they are evolutionarily conserved in land plants and are critical for germ cell differentiation and embryogenesis [11, 12, 19–23]. Ectopic expression of *RKD* genes in somatic cells induce cellular proliferation and, ultimately, the formation of somatic embryos [11, 12]. However, the precise molecular mechanisms implicated in this developmental reprogramming remain poorly understood.

In this study we found that in a black rice landrace (Oryza sativa L. cv. Cempo Ireng*),* the ectopic expression of *OsRKD3,* a gene preferentially expressed in reproductive tissues and during in vitro tissue culture, induces the formation of somatic embryos. How de-differentiation, organogenesis, and SE are induced by RKD transcription factors is not yet known, however, our data support the view that RKD transcription factors lie upstream of a transcriptional cascade implicated in totipotency in plants. This idea is supported by the presence of *OsRKD3* binding motifs in upstream regulatory regions of several AP2/ERF genes that are activated in response to *OsRKD3* expression. Similarly, *Arabidopsis* RKD4 (AtRKD4) activate the expression of *AP2/ERF* genes in somatic tissues [11]. Several reports have shown that AP2/ERF transcription factors such as BABY BOOM (BBM), AINTEGUMENTA (ANT) and PLETHORA (PLT) play a critical role in meristem homeostasis [44–46], are activated by exogenous phytohormones and are implicated in totipotency as they have the capacity to convert somatic cells to embryonic cells [6, 44, 47]. Similarly, the expression of *WOUND INDUCED DEDIFFERENTIATION 1* (*WIND1*) – an AP2/ERF transcription factor induced by wounding, has been implicated in SE as its ectopic expression increases de novo shoot regeneration from root explants [8, 37]. We also found that *OsRKD3* regulates the activity of a HOMEODOMAIN LEUCINE ZIPPER (*HD-Zip*) transcription factor. HD-Zips play a critical role in meristem and embryo development and are thus tightly regulated transcriptionally [48, 49] and post-transcriptionally [50].

In addition, the transcriptional cascade initiated by *OsRKD3* appears to result in the activation of an embryogenic program in somatic cells. In Arabidopsis, BBM activates the LEAFY COTYLEDON1 (*LEC1*), LEAFY COTYLEDON2 (*LEC2*), ABSCISIC ACID-INSENSITIVE3 (*ABI3*) and FUSCA3 (*FUS3*) network [51]. This network induces the expression of embryo-specific genes and in some cases somatic embryos [13, 14, 44, 51–54]. Notably, we did not find evidence for OsRKD3 activation of rice BBM genes, which have been shown to be implicated in somatic embryogenesis [47]. Ectopic overexpression of rice BBM1 (OsBBM1) can induce somatic embryogenesis on rice leaves even in the absence of auxin [44]. It is possible that OsBBM1 induces the initiation of somatic embryos in rice by promoting the activation of OsYUCCA (OsYUC) genes, which are known to be involved in the biosynthesis of auxin [55]. It is possible that the expression of BBM genes in our experiments is below the level of detection and/or that these genes are regulated through a different transcriptional pathway. Since OsRKD3 modulates the expression of components of wounding and Jasmonate-mediated responses, the OsRKD3 and OsBBM pathways may be partially distinct from each other. Wheat shoot regeneration involves a sequential transcriptional cascade, in which several core transcription factors (TFs), including AP2, ERF, HD-ZIP, DOF, G2-like, and NAC, are found to be enriched [16], similar to the signal transduction mediated by OsRKD3. During the early stage of regeneration, ERF, WOX, WIND, and DOF genes are enriched, whereas ARF, BBM, and NAC genes appear in the later stages of wheat regeneration [16]. The transcriptional cascade initiated by *OsRKD3* also results in the upregulation of a discrete number of protein kinases and phosphatases that may participate in the development of SEs. Notably, RKD4 is required for the activation of a MAP kinase signalling pathway that regulates early embryo development in Arabidopsis [30]. In addition, the expression of the SOMATIC EMBRYOGENESIS RECEPTOR KINASE1 (*SERK1*) has been shown to enhance embryonic competence in culture [7].

Our data has also revealed that *OsRKD3* is involved in the repression of genes implicated in vegetative development and floral transition. The repression of vegetative development by RKD transcription factors may be important to delineate germline and embryo development in plants [21] but also for the resetting of juvenility. Notably, RKD4 and LEC2 have been found to mediate the de novo activation of several miR156 genes during gametogenesis and embryogenesis to reset juvenility in Arabidopsis [56]. Recent studies in Arabidopsis have shown that the resetting of vegetative and floral signatures is mediated by embryonic transcription factors and the removal of epigenetic signatures [57–59]. Moreover, the removal of epigenetic marks is also critical for the acquisition of pluripotency and developmental reprogramming, which underpins SE and organogenesis in tissue cultures [60]. A such, the somatic expression of native or engineered embryonic transcription factor genes and/or chemical compounds that reset epigenetic signatures have been used to increase the production of clonal plants and plant transformation efficiency in model and crop species [9, 10, 61, 62].

## Conclusions

In sum, our data show that *OsRKD3* can be used as an efficient tool to enhance the transformation potential of rice cultivars that are recalcitrant to tissue culture manipulation. Novel strategies to increase the efficiency of plant transformation or clonal propagation will be of paramount importance to engineer the genome of other similar orphan crops for improved production.

## Methods

All methods complied with relevant institutional, national, and international guidelines and legislation.

### Plant materials

Black rice (*Oryza sativa* L. cv. Cempo Ireng) seeds were obtained from the Centre for Biotechnology, Yogyakarta, Indonesia. Seeds were soaked overnight in tap water before sowing on germination medium (soil: compost fertilizer; 3:1), and only healthy seeds were used for propagation. Twenty-one day-old seedlings were planted in soil with compost fertilizer and maintained in a greenhouse under tropical conditions (12 h light, 12 h dark) with 200 μmol m^−2^ s^−1^ light intensity (35 °C daytime, 20 °C night). Transgenic lines were selfed to propagate seeds and the progenies were screened by PCR to confirm transgene presence and determine the number of transgene insertions. Only lines displaying segregation ratios for single transgene insertions were selected for propagation and further analysis.

### Black rice seed sterilization and callus induction

Mature seeds of black rice (*Oryza sativa* L. cv. Cempo Ireng) were dehusked and sterilized by immersion in 70% ethanol for 1 min followed by soaking for 2 min in 10% (v/v) sodium hypochlorite (5.25% active chlorine) and washed three times with sterile water for 15 min in total. The seeds were blotted dry with a sterilized filter paper then transferred onto callus induction medium with the scutellum pointing upward and cultured at 37 °C in continuous light conditions. For callus induction and proliferation, and for Agrobacterium transformation we used the same procedures, media composition and concentration of phyrhormones as described by FA Susanto, P Wijayanti, AN Fauzia, RD Komalasari, TR Nuringtyas and YA Purwestri [63]. Plant regeneration of indOsRKD4 transgenic plants was carried out using media lacking external phytohoromones.

### Vector construction and *Agrobacterium*-mediated transformation

For generation of the inducible-OsRKD3 vector, we designed full-length CDS of OsRKD3 (*synOsRKD3*), codon-optimized for rice and chemically synthesized (IDT, Leuven, BE) (Table S11). The synthesised *synOsRKD3* was introduced into a pDONR207 vector (Invitrogen, USA) via BP recombination and subcloned by LR recombination in the two-component chemically inducible vector *pTA7002* [31]. The pTA7002-*synOsRKD3* vector, thereafter named indOsRKD3, was fully sequenced and the construct was introduced into *Agrobacterium tumefaciens* EHA105. Rice transformation was performed by the *Agrobacterium*-mediated co-cultivation method and culture media as previously described [63–65].

### Genotyping of transgenic calli and plants

Genomic DNA isolation of putative transformant calli was performed using the CTAB method [66]. PCR was used to detect the inserted gene in the genome with specific primers: *synOsRKD3-*Fwd and syn*OsRKD3-*Rev, and to detect HPT with primer pair: Hygromycin-Fwd and Hygromycin-Rev (Table S11). PCR cycling conditions included denaturing at 94 °C for 30 s, annealing at 55 °C for 30 s and extension at 72 °C for 60 s. PCR products were separated by electrophoresis using 2% agarose concentration in 1 × TBE buffer.

### Induction of *OsRKD3* for the induction of somatic embryos

Embryonic calli transformed with *Agrobacterium tumefaciens* EHA105 carrying the pTA7002-*synOsRKD3* construct were transferred to N6-Dex medium containing 20 μM Dexamethasone for five days to induce somatic embryo formation. Explants were transferred onto hormone-free MS medium for five days to monitor the formation of somatic embryos using a stereo microscope and documented using a digital camera.

### Scanning Electron Microscopy (SEM)

Scanning electron microscopy was performed according to A Uemura, N Yamaguchi, Y Xu, WY Wee, Y Ichihashi, T Suzuki, A Shibata, K Shirasu and T Ito [67]. Briefly, the samples were placed in fixation solution containing 45% (v/v) ethanol, 5% (v/v) formaldehyde and 5% (v/v) acetic acid overnight at room temperature. The samples were then dehydrated with a series ethanol and acetone solutions. Samples then subjected to critical drying with liquid CO_2_ by using a critical point dryer (EM CPD300; Leica Microsystems). The E-1010 sputter coater (Hitachi) was used to perform sample coating with gold before SEM imaging was performed using S-4700 SEM (Hitachi) with an accelerating voltage of 15 kV.

### Histological study of *OsRKD3* induced somatic embryogenesis

Histological analysis of the embryogenic calli and somatic embryos was performed according to N Boissot, M Valdez and E Guiderdoni [68]. The putative embryogenic calli transformants obtained from the SE induction media were fixed in FAA (formalin-acetic acid–ethanol) for 24 h. Then, samples were dehydrated in a graded ethanol series (70, 95 and 100%) for 1 h each and embedded in paraffin wax. The sample was cut into 9–12 µm thick sections and stained with PAS-Hematoxylin. Somatic embryos were observed using an OlympusIX51 light microscope and photographed with a digital camera.

### Tissue staining with Sudan Red 7B

Calli were dehydrated through an isopropanol series (20%, 40%, 60%, 30 min each), and incubated for 1 h with a 0.5% Sudan Red 7B solution in 60% isopropanol. Samples were hydrated through a reverse isopropanol series (60%, 40%, 20%, 30 min each) and washed three times with water (30 min each). Samples were observed with a stereomicroscope equipped with a digital camera. To quantify staining differences between samples, calli stained with Sudan Red 7B was also ground in 80% ethanol followed by centrifugation for 5 min at 5600xg and supernatant was used by measuring absorbance at 528 nm using a spectrometer (Perkin-Elmer Instruments, USA).

### DEX treatment and collection of the treated samples

Immature tissue (calli) of wild type and pTA7002-*synOsRKD3* (indOsRKD3) plants were incubated in 20 μM of DEX solution containing 0.01% (w/v) Tween-20 for 3 h. As a mock control, wild type and transgenic samples were incubated in solution containing 0.02% (w/v) DMSO and 0.01% (v/v) Tween-20. After treatment with DEX and mock solutions, the solutions were drained, and the samples blotted with paper towels, placed into Eppendorf tube, then frozen in liquid nitrogen and stored at -80 °C until use.

### Phylogenetic tree and protein alignments

Rice RKD protein sequences were obtained from the MSU TIGR database (http://rice.uga.edu/). *Arabidopsis* and *Marchantia* RKD protein sequences were obtained from the NCBI database. The alignment of RKD proteins was computed using MAFFT in Jalview [69]. A phylogenetic tree was constructed using MEGA7 [70] using the minimum evolution method [71]. The tree was drawn to scale, with branch lengths in the same units as those of the evolutionary distances used to infer the phylogenetic tree. The evolutionary distances were computed using the Poisson correction method [72] and quantified according to the number of amino acid substitutions per site. The ME tree was searched using the Close-Neighbour-Interchange (CNI) algorithm [73] using a search level of 1. The Neighbour-joining algorithm [74] was used to generate the initial tree. All positions containing gaps and missing data were eliminated, such that a total of 88 positions remained in the final dataset.

### RNA library construction and sequencing

RNAs for transcriptional profile by sequencing were extracted using the Direct-zol RNA miniprep kit (ZYMO Research, Cambridge) from two independent biological replicates. The extracted RNAs were quantified using Qubit HS kit (Invitrogen, UK) and quality checked using a Bioanalyzer 2100 (Agilent, UK). Total RNA libraries were prepared using the TruSeq RNA Sample Preparation Kit (Illumina, UK) and sequenced in single-end 150 base mode an Illumina NexSeq500 platform at the University of Warwick.

### RNA sequencing data and GO term enrichment

Sequencing reads were pre-processed using Trimmomatic v0.36 [75] to remove reads with either low quality scores, irregular GC content, short length or sequencing adapters present. Trimmed reads were then quality checked using FastQC v0.11.5 [76] and mapped to the *Oryza sativa ssp. Japonica* genome (release MSU 7.0) using HISAT2 v2.1.0 [77]. Alignments were sorted using Samtools v0.1.19 [78], counted using LiBiNorm v2.0 [79] and then imported into R Studio (version 1.2.5033). Libraries were then normalised using DESeq2 v1.24 [80] and significant differentially expressed genes (DEGs) were calculated using the combined criteria; p.adj < 0.001, lfcThreshold > 1. Candidate selection involved filtering the DEGs to those shared between both pairwise condition comparisons to *indOsRKD3* induced expression. Upregulated DEGs were then tested for GO term enrichment with AgriGO v2.0 [81] using a Fisher’s exact test under the following criteria; FDR under dependency < 0.05.

### RKD motif enrichment analyses and co-expression network visualization

Motif enrichment analysis was carried out using the binding motif identified for Arabidopsis RKD2 [34] and 500 bp upstream sequences of the genes found to be upregulated upon ectopic expression of OsRKD3. Promoter sequences were analyzed using HOMER v. 4.11 [82] using default parameters and compared to an equal number of 500 bp upstream sequences from a random gene dataset. De-novo motif enrichment results were used to select an appropriate transcription factor class implicated in the network. Upregulated DEGs with a RKD binding motif in their upstream sequence were identified with HOMER and selected for co-expression network construction. Genes co-expressed with OsRKD3 target candidates were selected using RiceFREND [83] for visualization under the combined criteria; Hierarchy = 3, MR Rank = 7. Coexpression values for these genes were imported into Cytoscape version 3.6.1 [84] and were visualized under a perfuse force directed layout according to MR Rank. Annotation and arrangement of nodes was carried out using Cytoscape and Adobe Illustrator (v24.0.2).

### Promoter activity by Luciferase assays

To construct a luciferase fused with *LOC_Os06g04870*/*OsHD-Zip-like* promoter (*pOsHDZip-like::LUC*) we selected the 768 bp upstream sequence. We chemically synthesized a promoter sequence (IDT, Leuven, BE) and cloned them into a promoter-less luciferase reporter vector. For the constitutive expression of *OsRKD3*, we cloned that syn*OsRKD3* fragment in a pUbi-Gateway (GW) vector via recombination. All constructs developed in this study were fully sequenced. To measure the activity of *pOsHDZip-like::LUC* in response to *OsRKD3* induction, *Arabidopsis* mesophyll protoplast were transfected with 2 µg *pUbi::synOsRKD3*, 2 µg *pOsHDZip-like::LUC* and 1 µg *pUbi10::ß-Glucuronidase (GUS)* (used as the internal control). For negative control, 2 µg *pUbi* empty vector was co-transfected with protoplasts instead of *pUbi::synOsRKD3*. The co-transfected protoplasts were incubated for 12 h at 20 °C in the dark. We added a 75 μM D-luciferin solution and incubated protoplasts for 1 h under dark conditions. After dark incubation, luciferase activity was monitored using a Photek camera (Photek Limited, UK). For normalisation, we measured GUS activity by adding 4-methylumbelliferyl-β-D-glucuronide (MUG) as substrate and monitored the fluorescence of 4-methylumbelliferone (MU) using a fluorometer. Isolation of mesophyll protoplasts, co-transfection of plasmids, luciferase assay and MUG assay were carried out as previously described [85].

## Supplementary Information

**Additional file 1:** Representative images of black rice (Oryza sativa L. cv. Cempo Ireng) wild-type and indOsRKD3 calli stained with Sudan Red 7B.

**Additional file 2:** Molecular analysis of black rice plants transformed with indOsRKD3.

**Additional file 3:** Heatmap showing the expression profile, as normalised FPKM, of OsRKD3-modulated genes in different rice organs.

**Additional file 4:** **Fig. S4.** Heatmaps showing the expression profile, as normalised FPKM, of OsRKD3-modulated transcription factors in different rice organs.

**Additional file 5:** Supporting Tables.

**Additional file 6:** Unprocessed gel images.

## Data Availability

Sequence data (RNA-seq) that support the findings of this study have been deposited in the European Nucleotide Archive (ENA) under accession code PRJNA670218.
