# Supplementary material for: RWP-RK Domain 3 (OsRKD3) induces somatic embryogenesis in black rice
Source: BMC Plant Biol. 2023 Apr 19;23:202. doi: 10.1186/s12870-023-04220-z (PMC10114336; doi:10.1186/s12870-023-04220-z)
Supplement: Supplementary file 1 — Additional file 1: Representative images of black rice (Oryza sativa L. cv. Cempo Ireng) wild-type and indOsRKD3 calli stained with Sudan Red 7B. [file 12870_2023_4220_MOESM1_ESM.pdf]

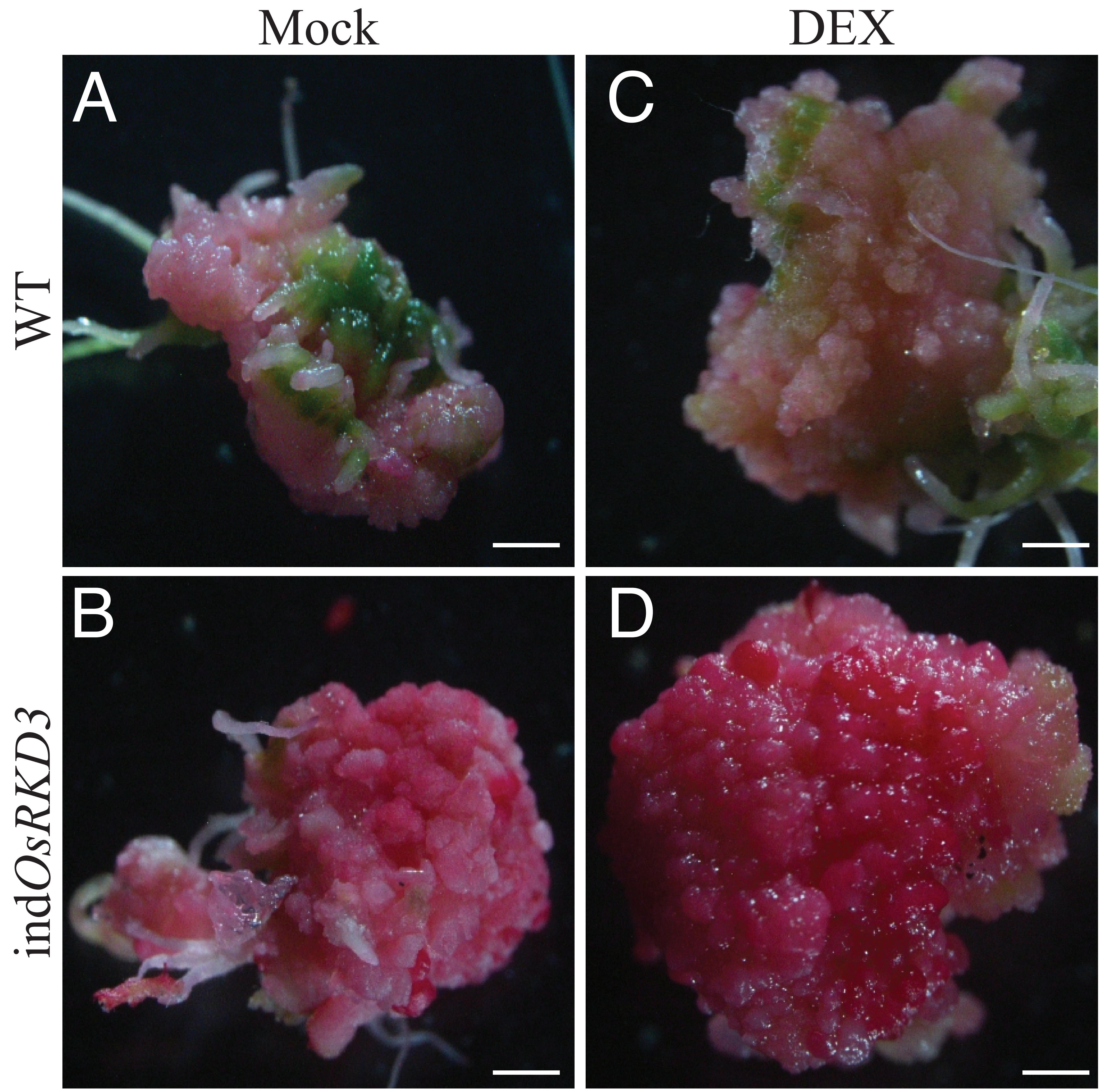

Supporting Figure S1. Representative images of black rice (Oryza sativa L. cv. Cempo Ireng) wild-type and indOsRKD3 calli stained with Sudan Red 7B. (A) Wild-type (WT) calli treated with a mock solution. (B) calli transformed with indOsRKD3 treated with a mock solution. (C) wild-type (WT) calli treated with a DEX solution. (D) calli transformed with indOsRKD3 treated with a DEX solution.
Only calli transformed with indOsRKD3 shows somatic embryo-like structures and a 6.3-fold Sudan Red 7B staining after treatment with a mock solution and 21.7-fold after DEX treatment (n= 6 calli with two biological replicates). Scale bars, 100 μm.
