# Supplementary material for: RWP-RK Domain 3 (OsRKD3) induces somatic embryogenesis in black rice
Source: BMC Plant Biol. 2023 Apr 19;23:202. doi: 10.1186/s12870-023-04220-z (PMC10114336; doi:10.1186/s12870-023-04220-z)
Supplement: Supplementary file 2 — Additional file 2: Molecular analysis of black rice plants transformed with indOsRKD3. [file 12870_2023_4220_MOESM2_ESM.pdf]

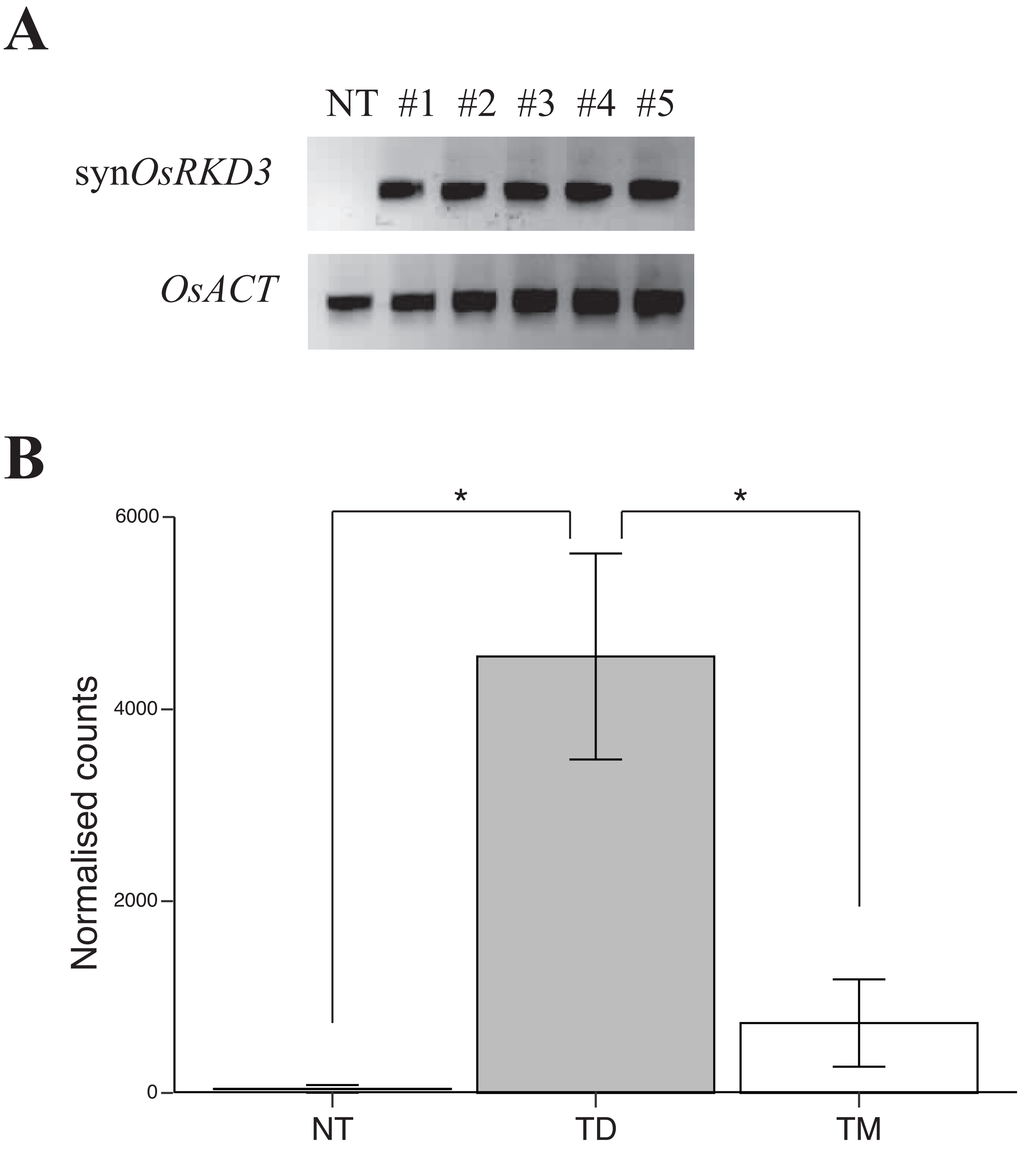


**Supporting Figure S2. Molecular analysis of black rice plants transformed with indOsRKD3.**
(A) PCR amplification of synOsRKD3 and Actin (OsACT) in independent transgenic lines.
(B) Bar plots of normalised RNA-seq read counts of synOsRKD3. ND, non-transgenic DEX treatment; TD, transgenic DEX treatment; TM, transgenic mock treatment. Error bars indicate standard deviations; n = 3. Tukey HSD post-hoc two-sided test * $p$-value $< 0.05$. .
