# Supplementary material for: RWP-RK Domain 3 (OsRKD3) induces somatic embryogenesis in black rice
Source: BMC Plant Biol. 2023 Apr 19;23:202. doi: 10.1186/s12870-023-04220-z (PMC10114336; doi:10.1186/s12870-023-04220-z)
Supplement: Supplementary file 3 — Additional file 3: Heatmap showing the expression profile, as normalised FPKM, of OsRKD3-modulated genes in different rice organs. [file 12870_2023_4220_MOESM3_ESM.pdf]

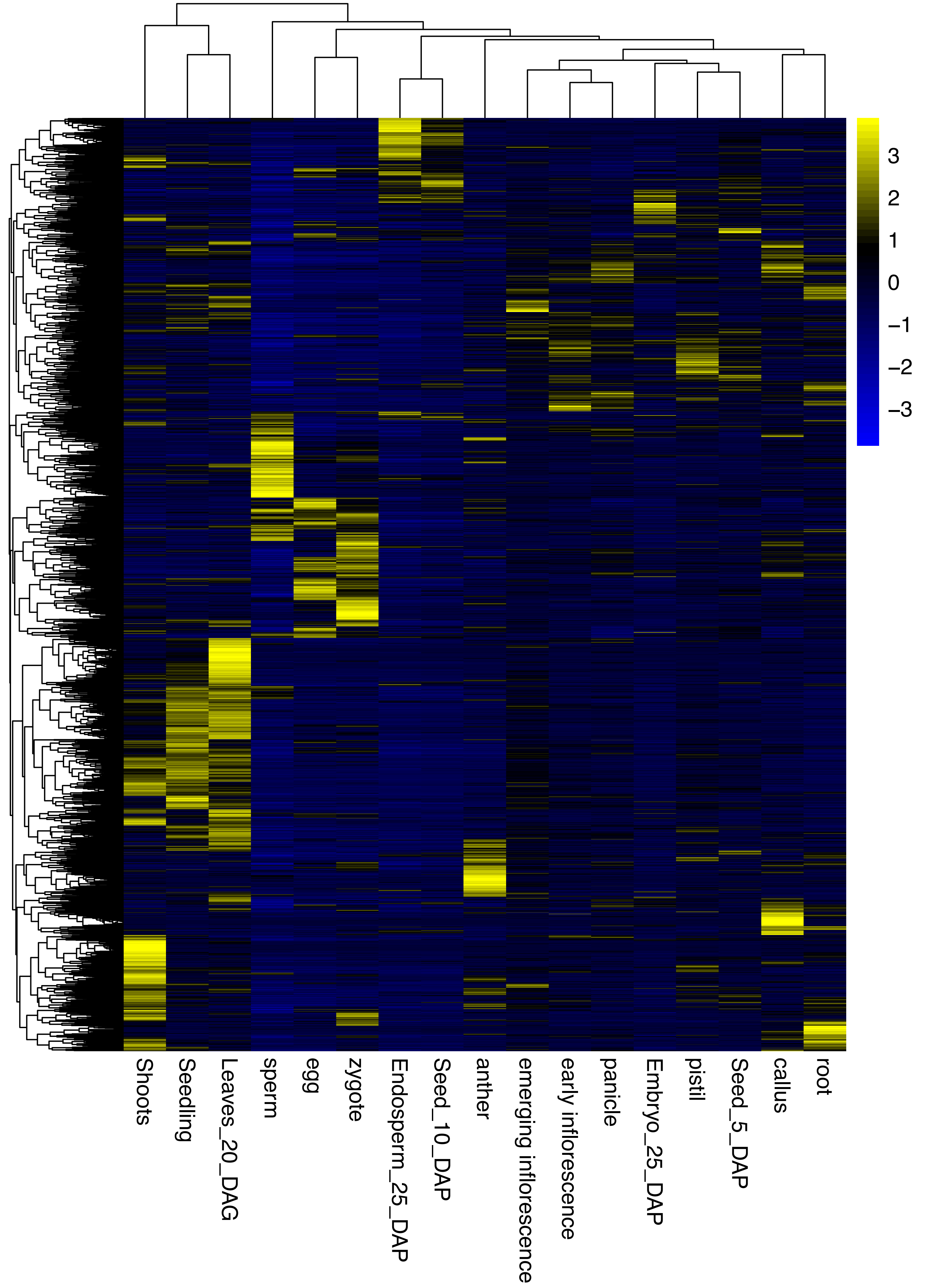

Supporting Figure S3. Heatmap showing the expression profile, as normalised FPKM, of OsRKD3-modulated genes in different rice organs.
