# Supplementary material for: RWP-RK Domain 3 (OsRKD3) induces somatic embryogenesis in black rice
Source: BMC Plant Biol. 2023 Apr 19;23:202. doi: 10.1186/s12870-023-04220-z (PMC10114336; doi:10.1186/s12870-023-04220-z)
Supplement: Supplementary file 4 — Additional file 4: Fig. S4. Heatmaps showing the expression profile, as normalised FPKM, of OsRKD3-modulated transcription factors in different rice organs. [file 12870_2023_4220_MOESM4_ESM.pdf]

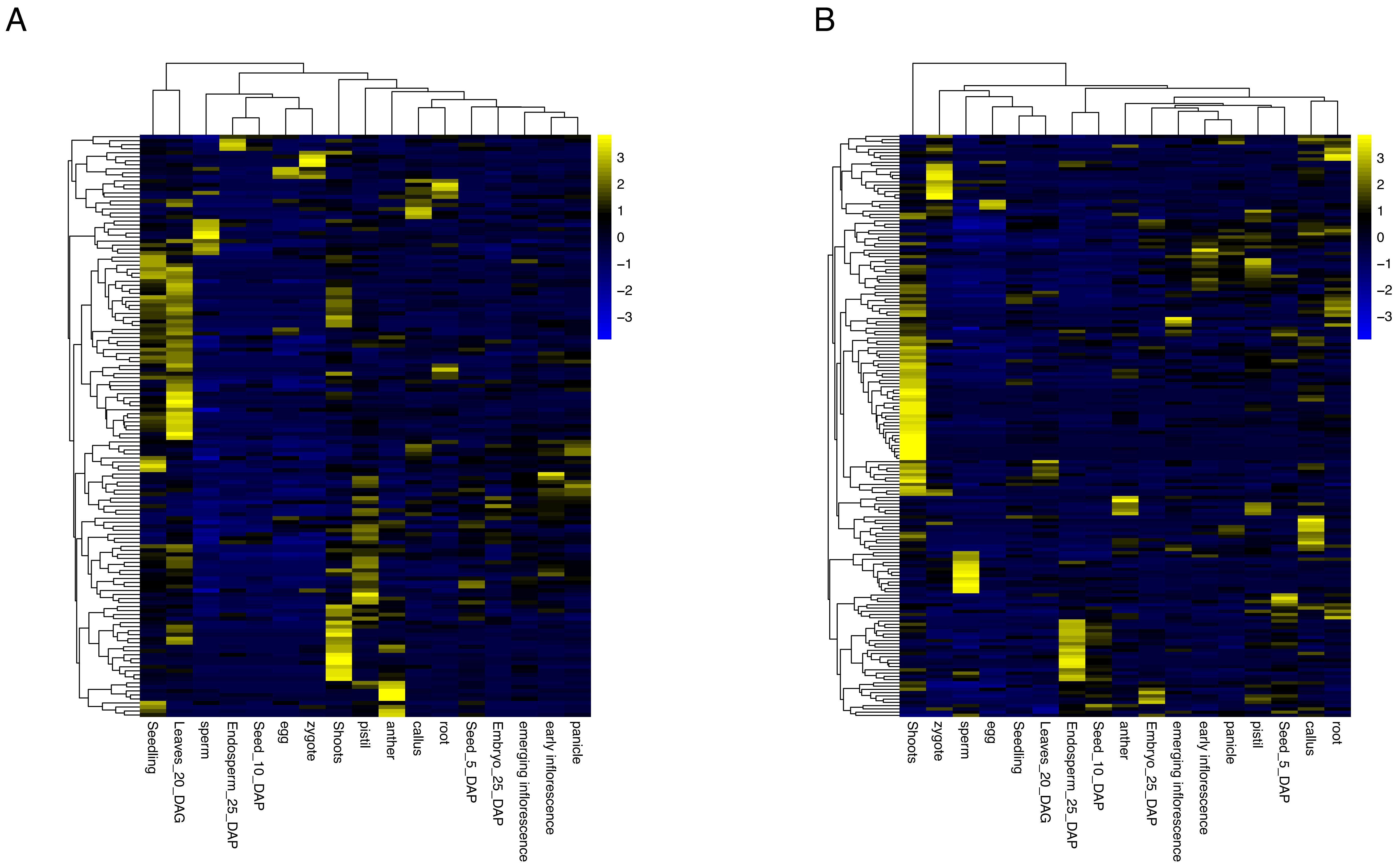

Supporting Figure S4. Heatmaps showing the expression profile, as normalised FPKM, of OsRKD3-modulated transcription factors in different rice organs.
(A) Expression profile of TFs upregulated upon ectopic expression of synOsRKD3. (B) Expression profile of TFs downregulated upon ectopic expression of synOsRKD3..
