# Supplementary figures and images for: RWP-RK Domain 3 (OsRKD3) induces somatic embryogenesis in black rice
Source: BMC Plant Biol. 2023 Apr 19;23:202. doi: 10.1186/s12870-023-04220-z (PMC10114336; doi:10.1186/s12870-023-04220-z)

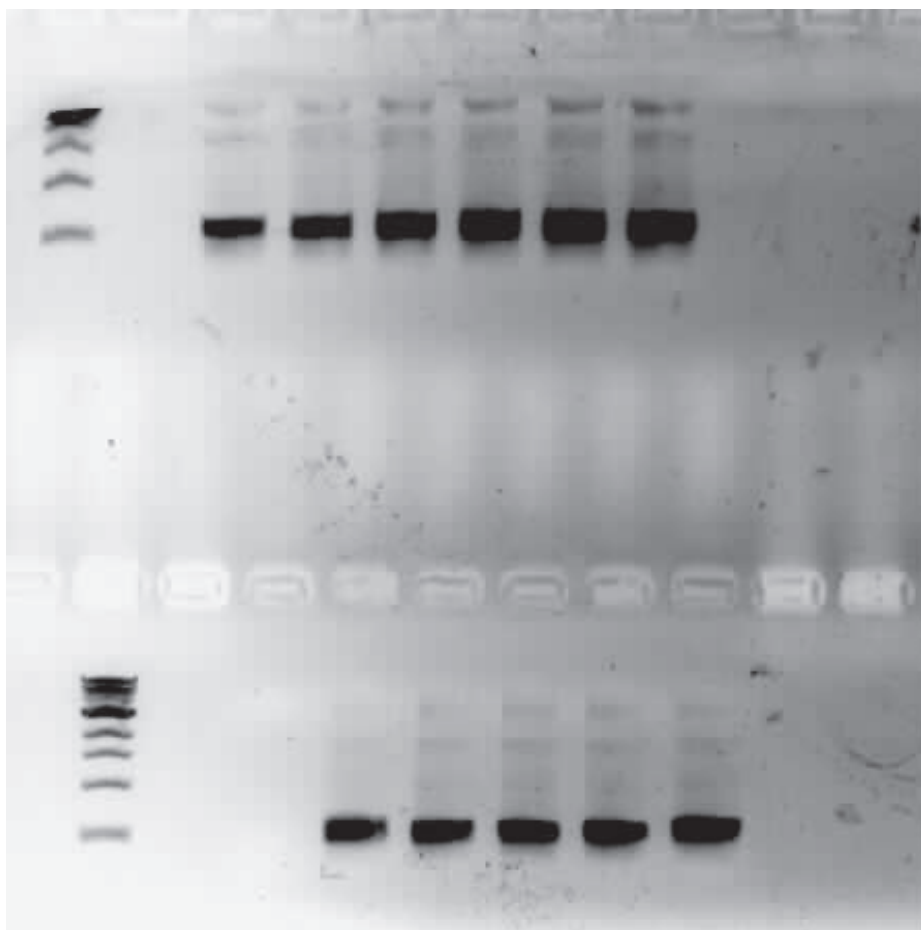

Unprocessed gel picture of Supplementary Figure 2

Supplement: Supplementary file 6 — Additional file 6: Unprocessed gel images. [file 12870_2023_4220_MOESM6_ESM.pdf]
